# Healthcare utilization among patients with rheumatoid arthritis, with and without herpes zoster, a retrospective administrative data linked cohort study

**DOI:** 10.1371/journal.pone.0323229

**Published:** 2025-05-13

**Authors:** Mohammad Movahedi, Angela Cesta, Xiuying Li, Mark Robert Tatangelo, Claire Bombardier, on behalf of OBRI investigators

**Affiliations:** 1 Toronto General Hospital Research Insitute, University Health Network, Toronto, Ontario, Canada; 2 Institute of Health Policy, Management, and Evaluation (IHPME), University of Toronto, Toronto, Ontario, Canada; 3 Department of Medicine, University of Toronto, Toronto, Ontario, Canada; Mayo Clinic College of Medicine and Science, UNITED STATES OF AMERICA

## Abstract

**Background:**

Herpes zoster (HZ) infection is a significant concern among seniors and immunosuppressed patients including those with rheumatoid arthritis (RA). We aimed to compare healthcare utilization (HCU) and mortality in RA patients with and without HZ.

**Methods:**

Patients from the Ontario Best Practices Research Initiative (OBRI) a clinical cohort (2008–2020) were linked to the Institute for Clinical Evaluative Sciences (ICES), a population health database. Each HZ patient was matched to four non-HZ patients based on sex, age, and HZ diagnosis date. The incidence of primary (HCU including hospitalization, Emergency Department (ED) visits, physician visits) and secondary (mortality and chronic clinical conditions) outcomes was calculated for each cohort, along with the impact of disease activity, patient-reported outcomes, and RA medication on these outcomes.

**Results:**

The study included 269 RA patients with and 1072 without HZ. At index date (HZ diagnosis) patients with HZ were less likely to have private health insurance (45.7% vs. 56.5%) and more prone to use biologics (30.9% vs. 26.8%) and JAK inhibitors (3.7% vs. 2.6%). Hospitalization/ED visits and mortality were higher in HZ patients, but these differences were not statistically significant after adjusting for other factors. HZ patients had significantly more physician visits (adj IRR: 1.17; 95% CI: 1.03–1.33). Female sex and lower CDAI were associated with fewer physician visits. JAK inhibitor use was associated with increased mortality (adj HR: 4.73, 95% CI: 1.68, 13.4).

**Conclusion:**

HCU was higher in RA patients with HZ, particularly in physician visits. Disease activity, patient reported outcomes and RA medication used did not have an impact on HCU and mortality.

## Introduction

Herpes Zoster (HZ) is common among seniors and especially concerning among patients who are elderly, female, and immunosuppressed. Immunosuppression caused by factors such as human immunodeficiency virus (HIV), acquired immune deficiency syndrome (AIDS), or cancer is one of several risk factors that can weaken immunity and raise the likelihood of HZ [1,2]. Comorbidities such as Rheumatoid Arthritis (RA), systemic lupus erythematosus, and inflammatory bowel disease are also associated with increased risk of HZ infection [1]. For example, patients with RA have approximately a twofold increased risk of developing HZ compared to the general population [2–5]. This elevated risk is attributed to RA disease itself, and related therapies (corticosteroids, biologic agents, Janus Kinase inhibitors (JAKi)) used to manage RA [3,6,7] which have an immunosuppressive effect on the patient.

Prior research on the burden of HZ have relied on population administrative datasets that capture the illness and related costs well but do not contain data on the clinical factors associated with burden of disease [2,8,9]. In contrast, clinical studies of HZ contain detailed clinical information but no accurate measurement of healthcare utilization and related costs [10,11]. Linking longitudinal clinical cohort data to population administrative datasets allows the opportunity to assess the association of disease activity, comorbid conditions, and clinical events on healthcare resource utilization (e.g., cost, serious adverse events).

We aimed to compare hospitalizations, emergency department (ED) visits, physician visits, and death in RA patients with and without HZ, adjusting for RA clinical data.

## Methods

We conducted a population-based longitudinal retrospective cohort study of adult RA patients, in Ontario, Canada, with newly diagnosed HZ between 01 January 2008 and 31 March 2020 matched to controls without HZ but the same age and sex at time of HZ diagnosis (index date) in cases.

### Ethic statement

All sites had received ethics approval to enroll patients, and all participants provided informed consent, Participants were required to be 18 years of age or older. The ethics approval reference number is REB# 07–0729, granted by the University Health Network.

For this study we accessed the Ontario Best Practices Research Initiative (OBRI) clinical data as of April 20, 2023 and transferred it to the Institute for Clinical Evaluative Sciences (ICES). Authors had no access to information that could identify individual participants during or after data collection. All data were fully anonymized before we accessed them.

### Study population

Patients with RA enrolled in the OBRI between 1^st^ Jan 2008 and 31 Dec 2020 were linked to the ICES administrative data. We included all adult (>=18 years) patients with RA that were diagnosed with HZ between Jan 1, 2008, and March 31, 2020 (RA with HZ). HZ was defined by the presence of an International Classification of Diseases, 9th Revision (ICD-9) [53] or ICD-10 [B02] or OHIP (53) code in the Discharge Abstract Database [12–14]. Only the first episode of HZ was considered as a study outcome in patients with multiple episodes during the study period.

**Fig 1:**
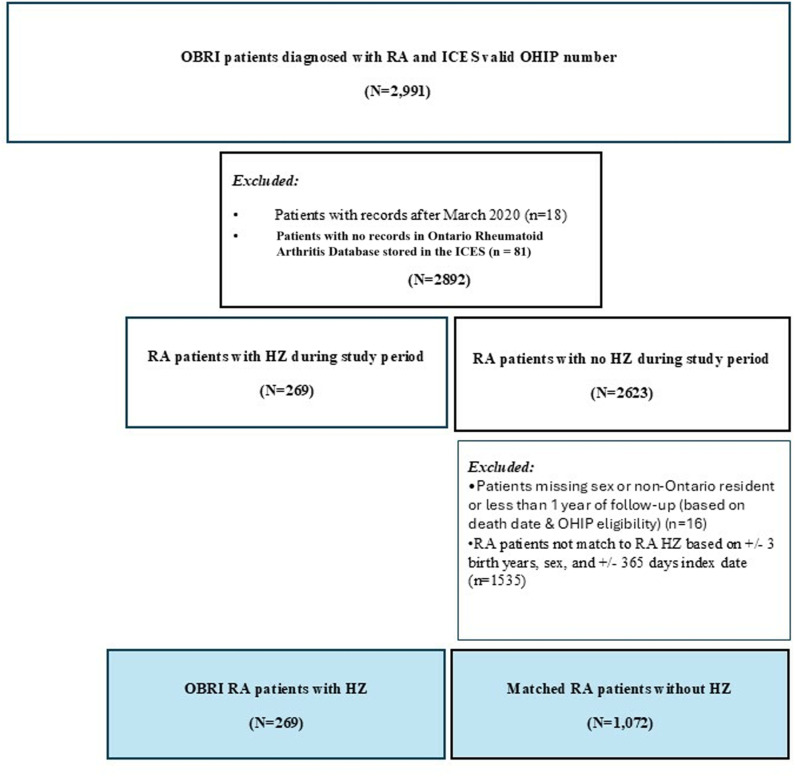
Cohort flowchart.

We excluded patients with missing or invalid Ontario Health Insurance Plan (OHIP) coverage (i.e., non-residents), those under 18 years of age, and those with less than 1-year follow-up (based on death date and OHIP eligibility)). Fig 1 illustrates the cohort flow chart. Initially, 2,991 patients were enrolled in the OBRI. Of these, 18 had records beyond March 2020 and 81 were not identified as RA patients in Ontario Rheumatoid Arthritis Database (ORAD) stored in the ICES; these patients were excluded. This left 2,892 patients, of whom 269 were diagnosed with HZ infection (case cohort). Among the 2,623 patients without HZ infection, 16 were excluded due to missing sex data, non-Ontario residency, or having less than one year of follow-up. Additional exclusions were made for patients who could not be matched to the RA with HZ cohort based on matching variables (n = 1,535). The final cohort consisted of 269 RA with HZ and 1,072 matched RA without HZ (Fig 1).

Each person was followed for up to 10 years from their index date, until death, or end of the study period (March 31, 2021), whichever occurred first.

### Data sources

**OBRI** is a multicentre RA registry established in 2008. The OBRI collects longitudinal data from both rheumatologists and patients across Ontario, from over 3,800 patients. It incorporates rheumatologist assessments from approximately one-third of the rheumatologists in Ontario. Enrolled patients are interviewed every 6 months by phone and are seen by their rheumatologist as per routine care. Data include clinical history, comorbidities, disease activity, inflammatory markers, tender and swollen joint counts, prior and current medication use, socio-demographics, work related questions, and functional status (Publications | Ontario Best Practices Research Initiative (obri.ca)).

At study enrollment and each subsequent visit, rheumatologists complete case report forms capturing key benchmark clinical covariates (Disease Activity Score-28 (DAS-28), Clinical Disease Activity Index (CDAI), tender/swollen joints, comorbid conditions) and current/past RA medication history. The patients are concurrently followed at regular 6-month intervals by trained telephone interviewers who conduct 20 minute interviews on a variety of patient reported outcomes (PROs) including patient quality of life (European Quality of Life (EQ5D-5L), work productivity, sleep quality, fatigue), functional status (Health Assessment Questionnaire (HAQ), Rheumatoid Arthritis Disease Activity Index [RADAI]), demographics (health insurance coverage, household annual income, rural residential status), and current medications. At study enrollment patients can consent to linking their data to Ontario’s Provincial repository of health data, held at ICES, using their unique OHIP number.

**ICES** is an independent, not-for-profit research institute whose legal status under Ontario’s health information privacy law allows them to collect and analyze health-related data without requiring patient consent. Records of publicly funded healthcare, for all residents with OHIP coverage, are captured in the ICES databases. The following ICES databases were used: Discharge Abstract Database (DAD), Same Day Surgery (SDS), National Ambulatory Care Reporting System (NACRS) for emergency department visits, OHIP for physician billings (diagnostic and fee codes), and the Registered Persons Database (RPDB), a population registry of vital statistics. The following ICES derived or acquired databases were also used: Chronic Obstructive Pulmonary Disease (COPD), Ontario Asthma Dataset (ASTHMA), Ontario Hypertension Dataset (HYPER), Congestive Heart Failure (CHF) database, Ontario Metal Health Reporting System (OMHRS), Ontario Diabetes Dataset (ODD), and the Ontario Cancer Registry (OCR). These datasets were linked using encrypted, unique health insurance numbers and were analyzed at ICES. Descriptions of databases and administrative codes are included in the supplements (Tables S1 and S2).

### Baseline sociodemographic and comorbidity profile

For the two cohorts, pre-existing comorbidities and patient demographics were determined using a two-year lookback period from the index date. Socio-demographic variables included age, sex, rural status [15], and neighborhood income quintile.

### Outcomes

HCU (including hospitalization admissions, ED visits, physician visits) was considered the primary outcome and death, and chronic clinical conditions as the secondary outcomes. Due to a small number of ED visits, we reported hospitalization and ED visits as a combined outcome. All physicians (family physicians and specialists) including inpatient and outpatient visits were included. Outcomes were measured form the index date up to 10 years of follow-up.

Chronic clinical conditions included Chronic Obstructive Pulmonary Disease (COPD), asthma, diabetes mellitus (DM), multiple sclerosis (MS), hypertension, Crohn and colitis, cystic fibrosis, HIV, dementia, Parkinson disease, chronic kidney disease (CKD), congestive heart failure (CHF), epilepsy, myasthenia gravis (MG), ischemic heart disease.

We identified chronic clinical conditions using validated case ascertainment algorithms for the following clinical conditions: COPD [16], asthma [17], DM [18], MS [19] hypertension [20], Crohn and colitis [21], dementia [22], Parkinson [23], CKD [24], CHF [25], epilepsy [26], MG [27], ischemic heart disease [28]. Where a validated algorithm did not exist, an algorithm similar to that used to derive the validated cohorts was used (i.e., at least one inpatient or two outpatient diagnoses within a two-year period) (Table S2).

### Statistical analysis

Each RA patient with HZ was matched to four RA patients without HZ. The control cohort was matched on sex, +/- 3 years of birth, and +/- 365 days of HZ infection (index date). By including more controls per case, researchers can improve the precision of their estimates and increase the power to detect associations between exposures and outcomes. This is especially useful when cases are limited in number [29]. Similarly, we chose window of +/- 3 years for birth date to increase number of controls matched to cases. Control groups without HZ were assigned a random index date based on the distribution of index dates from the case cohort. In cohort studies, random index dates can be used to match exposed individuals with unexposed ones. This ensures that the follow-up periods are similar, reducing bias in the comparison of outcomes.

Baseline characteristics were reported as means (standard deviation) for continuous variables and as numbers and percentages for categorical variables.

We used generalized linear mixed regression models (with negative binomial distribution) to calculate the crude incidence rate (IR) (per 100 person-years of clinical burden (hospitalizations, ED visits, physician visits, chronic clinical condition).

For death events, we used Cox regression models (from index date to death event) to estimate mortality rate (MR) in the RA with and without HZ cohorts. Proportionality assumption not violated based on martingale residual plots and supremum tests [30]. To calculate incidence rate ratio (IRR) and mortality rate ratio (MRR), the reference group was the RA cohort without HZ.

We additionally investigated the association between clinical disease activity, patient reported outcomes, RA medications and clinical burden using multivariable regression models.

IRR and MRR of outcomes of interest were estimated for the RA with HZ cohort (compared to RA without HZ) and other covariates (sociodemographic, clinical and medication characteristics) using multivariable models. All models were adjusted for a priori list of potential confounders including sex, age (log years were included as an offset in the models), disease duration, CDAI, RADAI, use of biologics, JAKi, conventional synthetic disease-modifying antirheumatic drugs (csDMARDs), and corticosteroids. Data on HZ vaccination was not available in ICES; therefore, we were not able to adjust for this variable.

A p-value of < 0.05 was considered statistically significant. In accordance with ICES data privacy policies, data for groups of five or fewer individuals was not included in the report to protect the privacy and confidentiality of individuals when reporting on sensitive or small population data.

All statistical models were run using SAS 9.4 statistical package [31].

## Results

### Baseline characteristics

We identified 269 RA patients diagnosed with HZ and 1,072 matched RA controls without HZ (Fig 1). Mean (SD) age was 63.7 (12.09) years for the RA with HZ cohort and 63.3 (12.03) years for the matched control cohort. A similar proportion of individuals lived in rural areas (11.9% vs. 12.6%) in both cohorts (Table 1). Patients with the lowest income were more susceptible to HZ infection (lowest income quintile: 19.0% vs. 16.2%). However, there was no specific trend for income quintiles and presence of HZ infection. Patients with HZ were less likely to have private health insurance coverage (45.7% vs. 56.5%). Compared to the RA without HZ, the RA with HZ cohort had lower disease activity (mean CDAI: 10.45 vs. 15.86) and were more likely to have a positive rheumatoid factor (RF) (70.3% vs. 64.1%). Patients with HZ used more biologic agents (30.9% vs. 26.8%) and JAKi (3.7% vs. 2.6%), less csDMARDs (59.2% vs. 87.2%), and less corticosteroids (17.1% vs. 25.6%) (Table 1).

**Table 1:** Baseline (RA diagnosis date) characteristics for RA cohorts with and without HZ.

Variable Label	Variable Value	RA with HZ (Cases) N = 269	RA without HZ (Controls) N = 1072
**Sex**	Female - n (%)	213 (79.2%)	849 (79.2%)
**Age**	Mean (SD)	63.74 (12.09)	63.33 (12.03)
**Age group**	18-44 - n (%)	22 (8.2%)	87 (8.1%)
	45-64 - n (%)	104 (38.7%)	443 (41.3%)
	65-84 - n (%)	135 (50.2%)	518 (48.3%)
	85 + - n (%)	8 (3.0%)	24 (2.2%)
**Nearest Census Based Neighborhood Income Quintile**			
	1 (Lowest) - n (%)	51 (19.0%)	174 (16.2%)
	2 - n (%)	40 (14.9%)	213 (19.9%)
	3 - n (%)	62 (23.0%)	200 (18.7%)
	4 - n (%)	45 (16.7%)	221 (20.6%)
	5 (Highest) - n (%)	71 (26.4%)	259 (24.2%)
**Residence**	Rural	32 (11.9%)	135 (12.6%)
**Health insurance coverage**	Private & public - n (%)	123 (45.7%)	606 (56.5%)
**Smoking status**	Current or past - n (%)	94 (34.9%)	508 (47.4%)
**Positive RF**	Yes - n (%)	189 (70.3%)	687 (64.1%)
**CDAI (0–76)**	Mean (SD)	10.45 (11.70)	15.86 (12.57)
**DAS28-ESR (0–9.4)**	Mean (SD)	3.10 (1.57)	3.77 (1.59)
**SDAI (0.1–86)**	Mean (SD)	11.85 (12.74)	17.30 (13.36)
**HAQ-DI (0–3)**	Mean (SD)	1.05 (0.80)	1.07 (0.75)
**Fatigue (0–10)**	Mean (SD)	4.38 (3.32)	4.29 (3.10)
**HAQ-pain (0–10)**	Mean (SD)	3.94 (2.99)	4.04 (2.83)
**RADAI (0–10)**	Mean (SD)	3.03 (2.31)	3.29 (2.12)
**Sleep problem (0–10)**	Mean (SD)	3.50 (3.30)	3.71 (3.34)
**Biologic agent use**	n (%)	83 (30.9%)	287 (26.8%)
**JAKi agent use**	n (%)	10 (3.7%)	28 (2.6%)
**csDMARDs use**	n (%)	159 (59.1%)	935 (87.2%)
**NSAIDs use**	n (%)	51 (19.0%)	206 (19.2%)
**Corticosteroids use**	n (%)	46 (17.1%)	274 (25.6%)

RA: rheumatoid arthritis; HZ: herpes zoster; RF: rheumatoid factor; CDAI: clinical disease activity index; DAS28-ESR: disease activity score 28-Erythrocyte sedimentation rate; SDAI: simplified disease activity score; HAQ-DI: health assessment questionnaire-disability index; RADAI: rheumatoid arthritis disease activity index; JAKi: Janus Kinase inhibitors; csDMARDs: conventional synthetic disease-modifying antirheumatic drugs; NSAIDs: nonsteroidal anti-inflammatory drugs.

### Healthcare utilization (HCU)

Overall, HCU was higher in the RA with HZ compared to the RA without HZ cohort.

There were 114 hospitalizations or ED visits among 269 patients with HZ, compared to 529 such events among 1072 patients without HZ (Table 2). Crude incidence rate of hospitalizations/ED visits were 72.86 (95% CI: 67.31–78.73) per 100 person-years in RA with HZ and 65.41 (95%CI: 62.99–67.90) per 100 person-years in RA without HZ. However, this difference was not statistically significant before (IRR: 1.172; 95%CI: 0.929–1.478) and after adjusting for disease duration, CDAI, RADAI, use of csDMARDs, biologic agents, JAKi, and corticosteroids at baseline (adj IRR: 1.137; 95%CI: 0.896–1.444) (Table 2). Female patients had a lower likelihood of hospitalizations/ED visits compared to male patients (adj IRR: 0.783; 95% CI: 0.634, 0.968), while older age and use of corticosteroids (adj IRR: 1.451; 95% CI: 1.191, 1.766) were associated with higher hospitalizations/ED visits (Table 2).

**Table 2:** Hospitalizations and emergency department visits in RA patients with and without HZ.

Covariates	Value	Crude Incidence (per 100 person-year)	Univariate Incidence Rate Ratio (95% CI) ¥	Multivariate Incidence Rate Ratio (95% CI) †¥
**Herpes Zoster infection**	RA without HZ N = 269 Events: 114	65.41 (62.99, 67.9)	Reference	Reference
	RA with HZ N = 1072 Events: 529	72.86 (67.31, 78.73)	1.172 (0.929, 1.478)	1.137 (0.896, 1.444)
**Sex**	Male	75.43 (70.27, 80.87)	Reference	Reference
	Female	64.41 (61.96, 66.94)	0.823 (0.666, 1.018)	**0.783 (0.634, 0.968)**
**Age (years)**	18-44	29.08 (24.22, 34.62)	Reference	Reference
	45-64	56.35 (53.23, 59.61)	1.934 (1.354, 2.761)	**1.848 (1.292, 2.643)**
	65-84	80.57 (77.02, 84.25)	2.924 (2.057, 4.156)	**2.834 (1.988, 4.039)**
	85+	128.24 (105.5, 154.43)	4.728 (2.493, 8.969)	**4.754 (2.521, 8.965)**
**Disease duration (years)**	<2 years	61.32 (57.46, 65.37)	Reference	Reference
	>=2 years	69.02 (66.31, 71.81)	1.14 (0.944, 1.377)	1.135 (0.929, 1.388)
**CDAI**	Non-LDA (>10)	69.44 (66.48, 72.5)	Reference	Reference
	LDA (<=10)	62.82 (59.48, 66.3)	0.845 (0.709, 1.006)	0.843 (0.697, 1.02)
**RADAI**	Non-LDA (>=2.2)	71.32 (68.47, 74.25)	Reference	Reference
	LDA (<2.2)	57.98 (54.48, 61.65)	0.802 (0.669, 0.961)	0.83 (0.682, 1.009)
**Use of biologics**	No	65.69 (63.07, 68.4)	Reference	Reference
	Yes	69.14 (64.96, 73.51)	1.09 (0.901, 1.319)	1.149 (0.943, 1.401)
**Use of JAKi**	No	66.81 (64.56, 69.12)	Reference	Reference
	Yes	60.4 (45.5, 78.62)	0.784 (0.463, 1.327)	0.93 (0.556, 1.555)
**Use of csDMARDs**	No	73.75 (67.54, 80.38)	Reference	Reference
	Yes	65.56 (63.18, 68)	0.907 (0.712, 1.156)	0.906 (0.71, 1.157)
**Use of Corticosteroids**	No	60.16 (57.74, 62.66)	Reference	Reference
	Yes	87.23 (82.08, 92.62)	1.518 (1.244, 1.853)	**1.451 (1.191, 1.766)**

**Notes:**

† N = 1,002 (RA patients with non-missing covariates)

¥ General linear mixed model (GLM) with negative binomial distribution

RA: rheumatoid arthritis; HZ: herpes zoster; CDAI: clinical disease activity index; LDA: low disease activity; RADAI: rheumatoid arthritis disease activity index; JAKi: Janus Kinase inhibitors; csDMARDs: conventional synthetic disease-modifying antirheumatic drugs

Among 269 patients with HZ, there were 163 physician visits, compared to 834 such events among 1072 patients without HZ (Table 3). Incidence rate of physician visits in RA with HZ was higher (2037.79 per 100 person- years) compared to RA without HZ (1934.16 per 100 person- years). After adjusting for other variables, physician visits remained significantly higher in the RA with HZ cohort (adj IRR: 1.167; 95% CI: 1.027–1.326) compared to RA without HZ (Table 3). Additionally, patients with low CDAI (adj IRR: 0.798; 95% CI: 0.722–0.883), and RADAI (adj IRR: 0.887; 95% CI: 0.800–0.984) were significantly less likely to visit their physician. In contrast, use of biologic agents (adj IRR: 1.163; 95% CI: 1.046–1.292) and corticosteroids (adj IRR: 1.215; 95% CI: 1.091–1.354) were significantly associated with a higher physician visit (Table 3).

**Table 3:** Physician visits in RA patients with and without HZ.

Covariates	Value	Crude Incidence Rate (per 100 person-year)	Univariate Incidence Rate Ratio (95% CI) ¥	Multivariate Incidence Rate Ratio (95% CI) †¥
**Herpes Zoster infection**	RA without HZ N = 269 Events: 163	1934.16 (1920.89, 1947.51)	Reference	Reference
	RA with HZ N = 1072 Events: 834	2037.79 (2008, 2067.91)	1.157 (1.016, 1.318)	**1.167 (1.027, 1.326)**
**Sex**	Male	1891.07 (1864.88, 1917.55)	Reference	Reference
	Female	1967.96 (1954.28, 1981.71)	0.899 (0.798, 1.013)	0.906 (0.809, 1.016)
**Age (years)**	18-44	1291.23 (1257.62, 1325.52)	Reference	Reference
	45-64	1762.92 (1745.25, 1780.73)	1.372 (1.143, 1.647)	**1.33 (1.113, 1.589)**
	65-84	2218.41 (2199.61, 2237.34)	1.966 (1.641, 2.355)	**1.927 (1.614, 2.3)**
	85+	2591.38 (2485.24, 2700.89)	2.286 (1.603, 3.26)	**2.306 (1.633, 3.256)**
**Disease duration (years)**	<2 years	1802.04 (1780.85, 1823.41)	Reference	Reference
	>=2 years	2016.92 (2002.16, 2031.76)	1.126 (1.015, 1.249)	1.066 (0.959, 1.184)
**CDAI**	Non-LDA (>10)	2059.5 (2043.21, 2075.88)	Reference	Reference
	LDA (<=10)	1800.45 (1782.37, 1818.66)	0.803 (0.73, 0.884)	**0.798 (0.722, 0.883)**
**RADAI**	Non-LDA (>=2.2)	2044.03 (2028.68, 2059.47)	Reference	Reference
	LDA (<2.2)	1778.54 (1758.89, 1798.36)	0.848 (0.768, 0.937)	**0.887 (0.8, 0.984)**
**Use of biologics**	No	1900.57 (1886.35, 1914.86)	Reference	Reference
	Yes	2077.51 (2054.32, 2100.88)	1.161 (1.044, 1.29)	**1.163 (1.046, 1.292)**
**Use of JAK inhibitors**	No	1958.85 (1946.58, 1971.17)	Reference	Reference
	Yes	1579.17 (1498.6, 1662.96)	0.692 (0.527, 0.909)	0.825 (0.635, 1.07)
**Use of csDMARDs**	No	2012.06 (1979.05, 2045.48)	Reference	Reference
	Yes	1942.38 (1929.34, 1955.48)	1.002 (0.876, 1.147)	0.979 (0.859, 1.115)
**Use of Corticosteroids**	No	1870.32 (1856.69, 1884.03)	Reference	Reference
	Yes	2208.83 (2182.59, 2235.3)	1.283 (1.146, 1.436)	**1.215 (1.091, 1.354)**

**Notes:**

† N = 1,002 (RA patients with non-missing covariates)

¥ General linear mixed model (GLM) with negative binomial distribution

RA: rheumatoid arthritis; HZ: herpes zoster; CDAI: clinical disease activity index; LDA: low disease activity; RADAI: rheumatoid arthritis disease activity index; JAKi: Janus Kinase inhibitors; csDMARDs: conventional synthetic disease-modifying antirheumatic drugs

### Death and chronic clinical conditions

Table 4 shows the death events in RA patients with and without HZ, unadjusted and adjusted for other variables. During the follow-up period, 18 out of 269 RA patients with HZ and 82 out of 1,072 RA patients without HZ died. There was no significant difference in mortality between the two cohorts (2.06 and 1.95 per 100 person-year in RA with HZ and without HZ, respectively). Even after making adjustments, there was no substantial difference in death events between the two cohorts (adj HR: 1.049; 95% CI: 0.612–1.800).

**Table 4:** Death events in RA patients with and without HZ.

Covariates	Value	Crude Mortality Rate (per 100 person-year)	Univariate Mortality Hazard Ratio (95% CI) ¥	Multivariate Mortality Hazard Ratio (95% CI)†¥
**Herpes Zoster infection**	RA without HZ N = 269 Events: 18	1.95 (1.55, 2.42)	Reference	Reference
	RA with HZ N = 1072 Events: 82	2.06 (1.22, 3.25)	1.019 (0.611, 1.697)	1.049 (0.612, 1.800)
**Sex**	Male	3.04 (2.08, 4.3)	Reference	Reference
	Female	1.69 (1.31, 2.14)	0.562 (0.369, 0.856)	0.662 (0.425, 1.032)
**Age (years)**	18-44	*	Reference	Reference
	45-64	0.74 (0.42, 1.2)	1.633 (0.375, 7.104)	1.611 (0.369, 7.030)
	65-84	3.21 (2.53, 4.01)	7.588 (1.863, 30.91)	**7.282 (1.778, 29.82)**
	85+	*	16.561 (3.193, 85.89)	**17.026 (3.258, 88.97)**
**Disease duration (years)**	<2 years	2.02 (1.37, 2.87)	Reference	Reference
	>=2 years	1.95 (1.51, 2.46)	0.966 (0.632, 1.476)	1.026 (0.642, 1.640)
**CDAI**	Non-LDA (>10)	2.22 (1.72, 2.83)	Reference	Reference
	LDA (<=10)	1.61 (1.12, 2.26)	0.768 (0.508, 1.163)	**0.616 (0.390, 0.973)**
**RADAI**	Non-LDA (>=2.2)	1.81 (1.38, 2.33)	Reference	Reference
	LDA (<2.2)	2.27 (1.62, 3.1)	1.313 (0.880, 1.959)	1.364 (0.888, 2.093)
**Use of biologics**	No	2 (1.56, 2.52)	Reference	Reference
	Yes	1.9 (1.26, 2.74)	0.953 (0.615, 1.474)	1.165 (0.720, 1.883)
**Use of JAK inhibitors**	No	*	Reference	Reference
	Yes	*	3.308 (1.205, 9.082)	**4.732 (1.675, 13.37)**
**Use of csDMARDs**	No	2.27 (1.3, 3.69)	Reference	Reference
	Yes	1.92 (1.53, 2.38)	0.813 (0.476, 1.389)	0.638 (0.365, 1.114)
**Use of Corticosteroids**	No	1.71 (1.33, 2.18)	Reference	Reference
	Yes	2.77 (1.92, 3.88)	1.585 (1.048, 2.398)	1.487 (0.961, 2.303)

**Notes:**

† N = 1,002 (RA patients with non-missing covariates)

¥ Cox proportional hazard model - proportionality assumption not violated based on Martingale residual plots and supremum tests

* Exact counts suppressed for privacy reasons. This applies to the whole table

RA: rheumatoid arthritis; HZ: herpes zoster; CDAI: clinical disease activity index; LDA: low disease activity; RADAI: rheumatoid arthritis disease activity index; JAKi: Janus Kinase inhibitors; csDMARDs: conventional synthetic disease-modifying antirheumatic drugs

The number of deaths were higher in older patients and those who used JAKi (adj HR: 4.732; 95% CI: 1.675, 13.37) across RA with and without HZ cohorts. In contrast, lower disease activity (adj HR: 0.616; 95%CI: 0.390–0.973) was significantly associated with a lower number of death (Table 4).

The incidence rate of chronic clinical conditions was numerically higher in RA with HZ (7.99 per 100 person-years) compared to RA without HZ (6.64 per 100 person-years). After adjusting for other variables, there was no significant difference between the RA with and without HZ cohorts (adj HR: 1.158; 95% CI: 0.84, 1.595).

Female patients had a lower likelihood of chronic clinical condition compared to male patients (adj IRR: 0.674; 95% CI: 0.508, 0.894). Compared to the 18–44 age group, the likelihood of chronic clinical conditions was higher in age groups 45–64 (adj IRR: 5.898; 95% CI: 2.123–16.384), 65–84 (adj IRR: 8.953; 95% CI: 3.241–24.734), and older than 85 (adj IRR: 12.622 95% CI: 3.680–43.292) (Table S3). No significant association was found between RA clinical and therapeutic class profiles and the likelihood of chronic clinical conditions.

## Discussion

HCU in RA patients with and without HZ was assessed using OBRI clinical data linked to provincial administrative healthcare data

We found that RA patients with HZ had numerically higher HCU including hospitalizations and ED visits compared to RA patients without HZ, even after accounting for patient’s clinical and therapeutic class profiles. However, the difference was statistically significant only for physician visits, with an adjusted incidence rate ratio of 1.167 (95% CI: 1.027, 1.326).

These findings are in line with other studies [5,32,33]. Singer et al showed that following a HZ diagnosis, all-cause healthcare utilization was higher in RA patients with HZ (N = 1,866) compared to RA patients without HZ (n = 38,846) [5]. They showed that hospitalizations and ED visits occurred more often in the RA with HZ cohort with an adjusted rate ratio of 1.16 (95% CI: 1.04, 1.30) for hospitalizations and 1.34 (95% CI: 1.21, 1.47) for ED visits. The limited number of hospitalizations and ED visits in our small study population could potentially explain the lack of statistically significant differences found in hospitalizations and ED visits between the two cohorts, as compared to the study by Singer et al.

Poetra et al also showed that the proportion of hospitalizations was 0.1% in RA patients with HZ compared to 0.04% in RA patients without HZ [32]. Johnson et al showed immunocompetent population with HZ had significantly higher healthcare utilization (inpatient visits, ED visits, outpatient visits, and other outpatient services) compared to immunocompetent patients without HZ [33].

We found the incidence rate of chronic clinical conditions were numerically higher in the RA with HZ cohort compared to the RA without HZ. However, the difference was not significant between the RA with and without HZ cohorts, suggesting HZ may not have a large impact on these particular chronic conditions. The small study population could potentially be another explanation for the lack of statistically significant differences.

We found similar patterns for death events with no significant differences between RA with and without HZ. This is also in line with other studies [34,35].

As expected, we also found that female patients had a lower likelihood of hospitalization and ED visits while hospitalization and ED visits increased with age.

Low CDAI and RADAI scores were associated with lower rate of hospitalizations, ED visits, death, and physician visits. These findings are in agreement with previous studies [36,37]. Curtis et al showed that there is a dose response association between disease activity (CDAI) and rate of hospitalizations, ED visits, and mortality [37]. This association was also shown for disease activity and risk of hospitalization due to infection [36].

In contrast, using biologic agents and corticosteroids were significantly associated with a higher physician visit. This finding is in agreement with a study showing that RA patients utilized more outpatient resources after undertaking biologic treatment [38].

We also found that death event was not higher in biologic or csDMARDs users. Listing et al showed that there was significantly lower mortality in patients treated with TNFi (adj HR = 0.64 (95% CI 0.50 to 0.81), rituximab (adj HR = 0.57 (95% CI 0.39 to 0.84), or other biologics (adj HR = 0.64 (95% CI 0.42 to 0.99), compared to those receiving methotrexate [39]. However, in this study we showed that use of JAK inhibitors is associated with a higher morality rate. The post-marketing safety surveillance also showed an increase in risk of death with 5mg (adj HR: 1.49; 95% CI: 0.80–2.74) and 10mg (adj HR: 2.39; 95% CI: 1.34–4.18) twice daily doses of tofacitinib in RA patients. The increased risk was only significant for 10 mg twice daily dose [40]. Further real-world studies on the impact of JAK inhibitors on mortality are required to confirm our results.

There are some limitations in our study. Misclassification of some RA patients may have affected the association between RA diagnosis and study outcomes. While we matched the cohorts for three main variables (age, sex, and date of HZ infection) and adjusted for certain clinical confounders, the possibility of residual confounders remains. Additionally, our findings are based on a Canadian population with a relatively small sample size, which might limit their external validation and generalizability to other healthcare systems. Another limitation of our study is the lack of data on HZ vaccinations. OHIP does not cover the cost of HZ vaccinations in Ontario, and because ICES databases only hold data for publicly funded healthcare costs, HZ vaccination status was not available in the ICES databases. In light of this, the OBRI has recently started collecting vaccination data from RA patients. This data may allow for a more comprehensive look at the impact of HZ vaccinations on HCU in RA patients.

We assume that the impact of misclassification would be small as we used the Ontario Rheumatoid Arthritis database (ORAD) to validate ICES-OBRI linked RA patients. The ORAD has been created using a validated algorithm [41].

Based on available knowledge, there is limited research on the impact of HZ on HCU specifically considering clinical data from RA patients. Notably, this study incorporated data from both administrative databases and our clinical registry to account for the influence of clinical characteristics and medication profiles. The aim was to explore the association between HZ infection and differences in HCU incidences among RA patients.

In conclusion, using population-based health administrative databases in Ontario, we found that while overall HCU was higher in RA patients with HZ compared to RA patients without HZ, only physician visits were shown to have significantly increased in RA patients with HZ. Disease activity, patient reported outcomes and therapeutic class use did not appear to have a significant impact on HCU and mortality in RA patients with HZ. Further research, with access to vaccination records and larger sample sizes, is required to better understand the association between clinical variables and HCU and mortality in this population.

**Key messages**

- Prior research on the burden of Herpes Zoster (HZ) has relied on Rheumatoid Arthritis (RA) population administrative datasets that capture the illness and related costs well but do not contain data on the clinical factors associated with burden of disease.While overall HCU (defined as hospitalizations, ED visits and physician) was higher in RA patients with HZ, only the number of physician visits was found to have significantly increased in the patients with HZ compared to those without HZ.
- While overall HCU (defined as hospitalizations, ED visits and physician) was higher in RA patients with HZ, only the number of physician visits was found to have significantly increased in the patients with HZ compared to those without HZ.
- Except corticosteroid use, no other clinical profiles were associated with higher hospitalization/ED visits, may be due to the small sample sizes in this study.
- Higher mortality was not found to be associated with HZ in RA patients, but the use of JAK inhibitors was associated with higher mortality, independent of HZ infection, warranting further study in real world settings.

## Supporting information

Table S1 ICES Data Sources used for this study. (DOCX)

Table S2 Definition of clinical conditions. (DOCX)

Table S3 Chronic clinical conditions^‡^ in RA patients with and without HZ. (DOCX)
