# Supplementary material for: Healthcare utilization among patients with rheumatoid arthritis, with and without herpes zoster, a retrospective administrative data linked cohort study
Source: PLoS One. 2025 May 13;20(5):e0323229. doi: 10.1371/journal.pone.0323229 (PMC12074599; doi:10.1371/journal.pone.0323229)
Supplement: Table S1 — (DOCX) [file pone.0323229.s001.docx]

## **Table S1.**

| **Ontario Health Insurance Plan Claims Database (OHIP)** |
| --- |
| The OHIP claims database contains information on inpatient and outpatient services provided to Ontario residents eligible for the province’s publicly funded health insurance system by fee-for-service health care practitioners (primarily physicians) and “shadow billings” for those paid through non-fee-for-service payment plans. The main data elements include patient and physician identifiers (encrypted), code for service provided, date of service, associated diagnosis, and fee paid. |
| **Discharge Abstract Database** |
| The DAD is compiled by the Canadian Institute for Health Information and contains administrative, clinical (diagnoses and procedures/interventions), demographic, and administrative information for all admissions to acute care hospitals, rehab, chronic, and day surgery institutions in Ontario. At ICES, consecutive DAD records are linked together to form ‘episodes of care’ among the hospitals to which patients have been transferred after their initial admission. |
| **Same Day Surgery Database (SDS)** |
| The SDS is compiled by the Canadian Institute for Health Information and contains administrative, clinical (diagnoses and procedures), demographic, and administrative information for all patient visits made to day surgery institutions in Ontario. The main data elements include patient demographics, clinical data (diagnoses, procedures, physician), administrative data (institution/hospital number etc.), financial data, service-specific data elements for day surgery and emergency. |
| **National Ambulatory Care Reporting System (NACRS)** |
| The NACRS is compiled by the Canadian Institute for Health Information and contains administrative, clinical (diagnoses and procedures), demographic, and administrative information for all patient visits made to hospital- and community-based ambulatory care centres (emergency departments, day surgery units, hemodialysis units, and cancer care clinics). At ICES, NACRS records are linked with other data sources (DAD, OMHRS) to identify transitions to other care settings, such as inpatient acute care or psychiatric care. |
| **Continuing Care Reporting System (for Chronic Care) (CCRS)** |
| The CCRS database is compiled by the Canadian Institute for Health Information and contains demographic, clinical, functional, and resource utilization information for individuals receiving facility-based continuing care (also known as extended, auxiliary, or complex chronic care) in Ontario hospitals and residential care providing 24-hour nursing services (i.e. nursing home). Clinical assessment data (on the physical, functional, cognitive, and social domains of health) is ascertained using the Resident Assessment Instrument Minimum Data Set (RAI-MDS) version 2.0 which is administered by trained healthcare professionals. |
| **National Rehabilitation Reporting System (NRS)** |
| The NRS is compiled by the Canadian Institute for Health Information and contains client data collected from participating adult inpatient rehabilitation facilities and programs across Canada. Main data elements contain socio-demographic information, administrative data (e.g. referral, admission, and discharge), health characteristics, activities, and participation (e.g. ADL, communication, social interaction), and interventions. |
| **Home Care Database (HCD)** |
| The HCD is a clinical client centric database that captures all services that are provided by or coordinated by Community Care Access Centres (CCACs). The data elements captured include information on client, intake, assessment, admission & discharge, diagnosis and surgical procedure, and care delivery. ICES receives home care data from the Ontario Ministry of Health and Long-Term Care (MOHLTC). The primary purpose of the information collected through the HCD is to aid in planning and better clinical insight into clients who encounter service through CCACs. |
| **Registered Persons Database files (RPDB)** |
| The RPDB provides basic demographic information (age, sex, location of residence, date of birth, and date of death for deceased individuals) for those issued an Ontario health insurance number. The RPDB also indicates the time periods for which an individual was eligible to receive publicly funded health insurance benefits and the best-known postal code for each registrant on July 1st of each year. |
| **Postal Code Conversion File (PCCF)** |
| The PCCF database will link to postal codes within a given cohort and determine other census geographic identifiers such as, dissemination/enumeration area, census division, longitude/latitude, urban/rural flag, and neighbourhood income quintile. |
| **Ontario Diabetes Dataset (ODD)** |
| The Ontario Diabetes Database is an ICES-derived cohort and is created using algorithms applied to inpatient hospitalization (DAD) records, same day surgery (SDS) records, and physician billing claims (OHIP) data to determine the diagnosis date for incident cases of diabetes in Ontario. For adults aged 19 years and greater, the definition for diabetes is 2 physician billing claims with a diagnosis for diabetes (OHIP diagnosis code: 250) or 1 inpatient hospitalization or same day surgery record with a diagnosis for diabetes (ICD-9 diagnosis code: 250; ICD-10 diagnosis codes: E10, E11, E13, E14; in any diagnostic code space) within a 2-year period. Physician claims and hospitalizations with a diagnosis of diabetes occurring within 120 prior to and 180 days after a gestational hospitalization record were excluded. |
| **Ontario Hypertension Dataset (HYPER)** |
| The Ontario Hypertension Database is an ICES-derived cohort and created using a definition of ≥2 physician billing claims with a diagnosis of hypertension (OHIP diagnosis codes: 401-405) and/or ≥1 inpatient hospitalization or same day surgery record with a diagnosis of hypertension (ICD-9 diagnosis codes: 401-405; ICD-10 diagnosis codes: I10-I13, I15; in any diagnostic code space) in a two-year period applied to hospitalization (DAD), same day surgery (SDS), and physician billing claims (OHIP) data to determine the diagnosis date for incident cases of hypertension in Ontario. Physician claims and hospitalizations with a diagnosis of hypertension occurring within 120 prior to and 180 days after a gestational hospitalization record are excluded. |
| **Ontario Congestive Heart Failure dataset (CHF)** |
| The Ontario Congestive Heart Failure Database is an ICES-derived cohort that was created using a definition of ≥2 physician billing claims with a diagnosis of CHF (OHIP diagnosis code: 428) and/or ≥1 inpatient hospitalization or same day surgery record with a diagnosis of CHF (ICD-9 diagnosis code: 428; ICD-10 diagnosis code: I50; in the primary diagnostic code space) in a two-year period applied to hospitalization (DAD), same day surgery (SDS), and physician billing claims (OHIP) data to determine the diagnosis date for incident cases of CHF in Ontario. |
| **Ontario Asthma Dataset (ASTHMA)** |
| The Ontario Asthma Database is an ICES-derived cohort that is created using a definition of ≥2 physician billing claims with a diagnosis of asthma (OHIP diagnosis code: 493) and/or ≥1 inpatient hospitalization or same day surgery record with a diagnosis of asthma (ICD-9 diagnosis code: 493; ICD-10 diagnosis codes: J45, J46; in any diagnostic code space) in a two-year period applied to hospitalization (DAD), same day surgery (SDS), and physician billing claims (OHIP) data to determine the diagnosis date for incident cases of asthma in Ontario. |
| **Ontario HIV Dataset (HIV)** |
| The Ontario HIV Database is an ICES-derived cohort that is created using a definition of ≥3 physician billing claims with a diagnosis of HIV (OHIP diagnosis codes: 042, 043, 044) in a three-year period applied to physician billing claims (OHIP) data to determine the diagnosis date for incident cases of HIV in Ontario. |
