# Supplementary material for: Healthcare utilization among patients with rheumatoid arthritis, with and without herpes zoster, a retrospective administrative data linked cohort study
Source: PLoS One. 2025 May 13;20(5):e0323229. doi: 10.1371/journal.pone.0323229 (PMC12074599; doi:10.1371/journal.pone.0323229)
Supplement: Table S2 — (DOCX) [file pone.0323229.s002.docx]

## **Table S2.**

| Condition | Algorithm | ICD-09 (CM) codes (Hospital codes) | ICD-10 codes (Hospital codes) | OHIP Dx codes |
| --- | --- | --- | --- | --- |
| Hypertension | One hospital admission with a hypertension diagnosis or an OHIP claim with a hypertension diagnosis followed within 2 years by either an OHIP claim or a hospital  admission with a hypertension diagnosis | 401x, 402x, 403x, 404x, 405x | I10, I11, I12, I13, I15 | 401, 402, 403, 404, 405 |
|  |  |  |  |  |
| Diabetes Mellitus | One hospital admission with a diabetes mellitus diagnosis or an OHIP claim followed within 2 years by either an OHIP claim or a hospital admission with diabetes mellitus diagnosis | 250 | E10, E11, E13, E14 | 250 |
|  |  |  |  |  |
| Multiple Sclerosis | One hospital diagnostic codes or outpatient physician billing codes (at least 5 from OHIP within a 2-year period) | 340 | G35 | 340 |
|  |  |  |  |  |
| Parkinson disease | 1 hospitalization record or 2 physician claim records at least 30 days apart in a 1-year period | 332.0, 332.1 | F0.3, G20, G210, G211, G212, G213, G214, G218, G219, G22 | 332 |
|  |  |  |  |  |
| Chronic Kidney disease | one hospitalization or 2 physician billings within a 2-year period | 403,585 | E102, E112, E132, E142, I12, I 13, N08, N18, N19 | 403,585 |
|  |  |  |  |  |
| Cardiovascular disease (CVD) | Includes myocardial infarction, ischemic heart disease, stroke, transit ischemic attack, congestive heart failure, other acute coronary syndrome | 410-414, 430-436, 362.3, 428 | I20-I25, I60-I64, H341, G45, I50 | 410-414, 430-436, 362, 428 |
|  |  |  |  |  |
| Ischemic heart disease | Two physician billings within a one-year period (with one of the billings by a specialist (spec in ('05' '09' '11' '12' '13' '17' '48' '60' '64') or a family physician (spec ='00') in a hospital or emergency room setting) or a hospital discharge abstract | 410-414 | I20-I25 | 410-414 |
|  |  |  |  |  |
|  |  |  |  |  |
| Atrial Fibrillation | One Hospital or emergency department diagnostic codes or outpatient physician billing codes (at least 4 from OHIP within a 1-year period) | 427.3 | I48 | 427.3 |
|  |  |  |  |  |
| Congestive heart failure | One hospitalization record, or one ambulatory record followed by a second record from either source within one year | 428 | I50,I50.1, I50.9 | 428 |
|  |  |  |  |  |
|  |  |  |  |  |
| Alzheimer’s and related Dementias | One hospitalization code OR (three physician claims codes at least 30 days apart in a two-year period) OR a prescription filled for an Alzheimer’s and related dementias specific medication. | 46.1, 290.0, 290.1, 290.2, 290.3, 290.4, 294.x, 331.0, 331.1, 331.5 | F00.x, F01.x, F02.x, F03.x, G30.x |  |
|  |  |  |  |  |
| Myasthenia gravis | 1 hospital discharge abstract with MG listed as a primary or secondary diagnosis, or 5 outpatient MG physician visits or 3 pyridostigmine prescriptions within 1 year | 358 | G70 | 358 |
|  |  |  |  |  |
| COPD | Age ≥35 and either: One OHIP claim with a COPD diagnosis or One hospital discharge or day surgery summary with a COPD diagnosis. | 491, 492, 496 | J41, J42, J43, J44 | 491, 492, 496 |
|  |  |  |  |  |
| Asthma | either: One hospital admission with an asthma diagnosis or Two OHIP claims with an asthma diagnosis within 2 years | 493 | J45, J46 | 493 |
|  |  |  |  |  |
| Herpes Zoster (HZ) | Any physician visit or an emergency department visit or hospital admission where the most responsible diagnosis  was herpes zoster. | 53 | B02 | 53 |
|  |  |  |  |  |
|  |  |  |  |  |
| Epilepsy | *For individuals 18 years and older:*  1 hospitalization record or 3 physician claim records at least 30 days apart in a 2-year period | 3450, 3451, 3454, 3455, 3456, 3457, 3458, 3459 | G40 | 345 |
| Cystic Fibrosis | At least one physician visit or an emergency department visit or hospital admission where diagnosis was cystic fibrosis. | 277 | E84 | 277 |
|  |  |  |  |  |
| Crohn and colitis | For Adults aged 18 to 64 years: at least 5 health contacts (outpatient physician visits or hospitalizations) within a 4-year window. Those who were 65 years or older required the addition of at least 1 prescription claim for an IBD-specific medication | 555.x, 556.x | K50.x, K51.x. | 555,556 |
|  |  |  |  |  |
|  |  |  |  |  |
