# Supplementary material for: Healthcare utilization among patients with rheumatoid arthritis, with and without herpes zoster, a retrospective administrative data linked cohort study
Source: PLoS One. 2025 May 13;20(5):e0323229. doi: 10.1371/journal.pone.0323229 (PMC12074599; doi:10.1371/journal.pone.0323229)
Supplement: Table S3 — (DOCX) [file pone.0323229.s003.docx]

## **Table S3.**

| Covariates | Value | Crude Incidence Rate (per 100 person-year) | Univariate Rate Incidence Ratio (95% CI) ^¥^ | Multivariate Incidence Rate Ratio (95% CI) ^†¥^ |
| --- | --- | --- | --- | --- |
| HZ infection | RA without HZ | 6.64 (5.88, 7.47) | Reference | Reference |
|  | RA with HZ | 7.99 (6.23, 10.1) | 1.231 (0.9, 1.682) | 1.158 (0.84, 1.595) |
|  |  |  |  |  |
| Sex | Male | 9.04 (7.31, 11.05) | Reference | Reference |
|  | Female | 6.31 (5.56, 7.14) | 0.679 (0.512, 0.899) | **0.674 (0.508, 0.894)** |
|  |  |  |  |  |
| Age (years) | 18-44 | * | Reference | Reference |
|  | 45-64 | 5.75 (4.78, 6.85) | 6.087 (2.194, 16.892) | **5.898 (2.123, 16.384)** |
|  | 65-84 | 8.79 (7.65, 10.07) | 9.476 (3.439, 26.113) | **8.953 (3.241, 24.734)** |
|  | 85+ | * | 12.68 (3.691, 43.564) | **12.622 (3.68, 43.292)** |
|  |  |  |  |  |
| Disease duration (years) | <2 years | 5.41 (4.31, 6.71) | Reference | Reference |
|  | >=2 years | 7.51 (6.63, 8.46) | 1.357 (1.024, 1.798) | 1.346 (0.998, 1.815) |
|  |  |  |  |  |
| CDAI | Non-LDA (>10) | 6.87 (5.96, 7.88) | Reference | Reference |
|  | LDA (<=10) | 6.89 (5.81, 8.1) | 0.98 (0.764, 1.257) | 0.944 (0.72, 1.237) |
|  |  |  |  |  |
| RADAI | Non-LDA (>=2.2) | 7.08 (6.21, 8.05) | Reference | Reference |
|  | LDA (<2.2) | 6.48 (5.35, 7.78) | 0.903 (0.696, 1.17) | 0.85 (0.646, 1.12) |
|  |  |  |  |  |
| Use of biologics | No | 6.97 (6.14, 7.89) | Reference | Reference |
|  | Yes | 6.64 (5.39, 8.09) | 0.949 (0.723, 1.245) | 0.971 (0.731, 1.292) |
|  |  |  |  |  |
| Use of JAKi | No | * | Reference | Reference |
|  | Yes | * | 0.753 (0.288, 1.967) | 0.808 (0.31, 2.107) |
|  |  |  |  |  |
| Use of csDMARDs | No | 7.25 (5.4, 9.53) | Reference | Reference |
|  | Yes | 6.81 (6.06, 7.63) | 0.936 (0.661, 1.324) | 0.894 (0.631, 1.266) |
|  |  |  |  |  |
| Use of Steroids | No | 6.67 (5.88, 7.54) | Reference | Reference |
|  | Yes | 7.51 (6.05, 9.21) | 1.145 (0.865, 1.517) | 1.1 (0.831, 1.455) |
|  |  |  |  |  |

**Notes:**

† N = 1,002 (RA patients with non-missing covariates)

¥ GLM with negative binomial distribution

‡ Chronic clinical events included: COPD, diabetes, multiple sclerosis, hypertension, Crohn and colitis, cystic fibrosis, HIV, dementia, Parkinson disease, chronic kidney disease, congestive heart failure, epilepsy, ischemic heart disease and myasthenia gravis.

RA: rheumatoid arthritis; HZ: herpes zoster; CDAI: clinical disease activity index; RADAI: rheumatoid arthritis disease activity index; JAKi: Janus Kinase inhibitors; csDMARDs: conventional synthetic disease-modifying antirheumatic drugs
